# Reversing painful and non-painful diabetic neuropathy with the capsaicin 8% patch: Clinical evidence for pain relief and restoration of function *via* nerve fiber regeneration

**DOI:** 10.3389/fneur.2022.998904

**Published:** 2022-10-26

**Authors:** Praveen Anand, Rosario Privitera, Philippe Donatien, Hassan Fadavi, Solomon Tesfaye, Vassiliki Bravis, V. Peter Misra

**Affiliations:** ^1^Division of Neurology, Hammersmith Hospital, Imperial College London, London, United Kingdom; ^2^Diabetes Research Unit, Sheffield Teaching Hospitals NHS Foundation Trust, Sheffield, United Kingdom; ^3^Department of Endocrinology and Diabetes, St Mary's Hospital, Imperial College Healthcare NHS Trust, London, United Kingdom

**Keywords:** capsaicin, diabetic neuropathy, pain, clinical trial, skin biopsy

## Abstract

**Introduction:**

Current oral treatments for pain in diabetic peripheral neuropathy (DPN) do not affect the progression of DPN i.e., “disease modification.” We assessed whether Capsaicin 8% patch treatment can provide pain relief and also restore nerve density and function *via* nerve regeneration, in both painful (PDPN) and non-painful (NPDPN) diabetic peripheral neuropathy.

**Methods:**

50 participants with PDPN were randomized to receive Capsaicin 8% patch Qutenza with Standard of Care (SOC) (PDPN Q+SOC group), or SOC alone (PDPN SOC group). Pain symptoms were assessed with a diary (Numerical Pain Rating Scale, NRPS) and questionnaires. Investigations included quantitative sensory testing (QST) and distal calf skin biopsies, at baseline and 3 months after baseline visit; subsequent options were 3-monthly visits over 1 year. 25 participants with NPDPN had tests at baseline, and 3 months after all received Capsaicin 8% patch treatment.

**Results:**

At 3 months after baseline, PDPN Q+SOC group had reduction in NPRS score (*p* = 0.0001), but not PDPN SOC group. Short-Form McGill Pain Questionnaire (SF-MPQ) showed significant reductions in scores for overall and other pain descriptors only in the PDPN Q+SOC group. Warm perception thresholds were significantly improved only in the PDPN Q+SOC group (*p* = 0.02), and correlated with reduction in SF-MPQ overall pain score (*p* = 0.04). NPDPN Q+SOC group did not report pain during the entire study. Density of intra-epidermal nerve fibers (IENF) with PGP9.5 was increased at 3 months in PDPN Q+SOC (*p* = 0.0002) and NPDPN Q+SOC (*p* = 0.002) groups, but not in the PDPN SOC group. Increased sub-epidermal nerve fibers (SENF) were observed with GAP43 (marker of regenerating nerve fibers) only in PDPN Q+SOC (*p* = 0.003) and NPDPN Q+SOC (*p* = 0.0005) groups. Pain relief in the PDPN Q+SOC group was correlated with the increased PGP9.5 IENF (*p* = 0.0008) and GAP43 (*p* = 0.004), whereas those with lack of pain relief showed no such increase; in some subjects pain relief and increased nerve fibers persisted over months. PGP9.5 IENF increase correlated with axon-reflex vasodilatation in a NPDPN Q+SOC subset (*p* = 0.006).

**Conclusions:**

Capsaicin 8% patch can provide pain relief *via* nerve regeneration and restoration of function in DPN (disease modification). It may thereby potentially prevent diabetic foot complications, including ulcers.

## Introduction

Painful diabetic peripheral neuropathy (PDPN) may be defined as pain caused by a lesion of the peripheral somatosensory system attributable to diabetes mellitus (DM). Chronic pain can be a severe clinical manifestation of diabetic peripheral neuropathy, with a prevalence ranging from 8–30% reported in several studies of long-standing DM (1). The underlying mechanisms leading to small fiber sensory polyneuropathy and the associated pain in diabetes are diverse (2–6).

Current treatments for treating neuropathic pain in patients with PDPN have limited efficacy and significant side effects (7). These include tricyclic antidepressants (e.g., amitriptyline), serotonin noradrenaline reuptake inhibitors (e.g., duloxetine and venlafaxine), pregabalin, and gabapentin. Other treatments are lidocaine patches, and topical capsaicin, including the capsaicin 8% patch (Qutenza) (8). At present, there are no disease-modifying therapies licensed in the EU or USA for PDPN or Non-Painful Diabetic Peripheral Neuropathy (NPDPN) (9).

Capsaicin 8% patch (Qutenza) is a TRPV1 receptor agonist therapy, with several advantages over the alternative traditional low-dose topical formulations. It has been used to treat peripheral neuropathic pain as licensed in the EU, including PDPN, and for treating post-herpetic neuralgia (PHN) and PDPN, as licensed in the USA. The evidence and mechanisms for pain relief by capsaicin 8% patch (Qutenza) have been reviewed (2, 10–13). Interestingly, pain relief may persist for months after a single 30-min capsaicin 8% patch application in PDPN, as reported in 1/3 subjects (14). Further, there is a report of progressive pain relief effect of 2-monthly repeated capsaicin 8% patch applications over a year, in a PDPN safety study (15, 16).

There is little data regarding the effect of topical treatment with capsaicin 8% patch (Qutenza) in skin biopsies. The time-course of nerve fibers degeneration and then regeneration over weeks following high dose topical capsaicin in skin biopsies have been reported by us in a human volunteer study (17), and by another group after capsaicin 8% patch application (18). Treatment with topical capsaicin in human experimental model studies have shown slower regeneration of intra-epidermal nerve fibers (IENFs) following their degeneration in patients with DM, particularly in those with DPN (19) or type 2 DM (20).

In clinical treatment combined with tissue studies, capsaicin 8% patch (Qutenza) application for post-herniorrhaphy neuropathic pain did not lead to significant pain relief or change of IENFs in skin biopsies from the site of surgery (21). In painful chemotherapy-induced peripheral neuropathy (CIPN), capsaicin 8% patch (Qutenza) application to the feet and distal calf led to a reduction of pain, along with a significant increase of intra-epidermal and sub-epidermal nerve fibers in distal calf skin biopsies, and improvement in other mechanistic biomarkers (22, 23). Capsaicin 8% patch (Qutenza) treatment in non-freezing cold injury (NFCI) also led to pain relief and nerve regeneration; importantly, pain relief correlated with restoration of nerve fibers (23), in support of our proposed mechanism of “disease-modification” (22, 23).

The aim of this study was to assess the mechanisms of pain relief following application(s) of the capsaicin 8% patch (Qutenza) in patients with Painful Diabetic Peripheral Neuropathy (PDPN). Patients with PDPN were randomized to receive capsaicin 8% patch (Qutenza) with Standard of Care, or Standard of Care alone. The patients were assessed at baseline and 3 months after baseline (thereafter 3-monthly as optional). The investigations included pain diary, questionnaires, quantitative sensory testing (QST), and skin biopsies studied with a range of biomarkers. Our findings led us to extend these assessments to patients with Non-Painful Diabetic Peripheral Neuropathy (NPDPN), in an open label single treatment study with capsaicin 8% patch.

We have reported progressive loss of nerve fibers over 6 months in skin biopsies collected from patients with longstanding DPN, in a natural history study (24). In this study, we have assessed the efficacy of capsaicin 8% patch (Qutenza) application for improving nerve regeneration and restoration of nerve fibers in patients with PDPN or NPDPN. Loss of sensory nerve fibers and their protective function are the major contributors to the development, recurrence or non-healing of foot ulcers, leading to amputations, for which the capsaicin 8% patch (Qutenza) could potentially provide preventative treatment (25–32).

## Materials and methods

### Study design

This was a single-center study on the effects of Capsaicin 8% patch treatment. Seventy-five subjects (*n* = 50 with Painful Diabetic Peripheral Neuropathy, and *n* = 25 with Non-Painful Diabetic Peripheral Neuropathy) participated in this study. Study approval was obtained from the East of England, Cambridgeshire and Hertfordshire Research Ethics Committee (Ethics reference number: 17/EE/0498), the European Union Drug Regulating Authorities (EudraCT Number: 2017-004746-17), and registered in the ISRCTN registry (https://doi.org/10.1186/ISRCTN14254122). The study was monitored by the Joint Research Compliance Office, Imperial College London and Imperial College Healthcare NHS Trust, in accord with Good Clinical Practice (GCP) guidelines.

**Figure 1:**
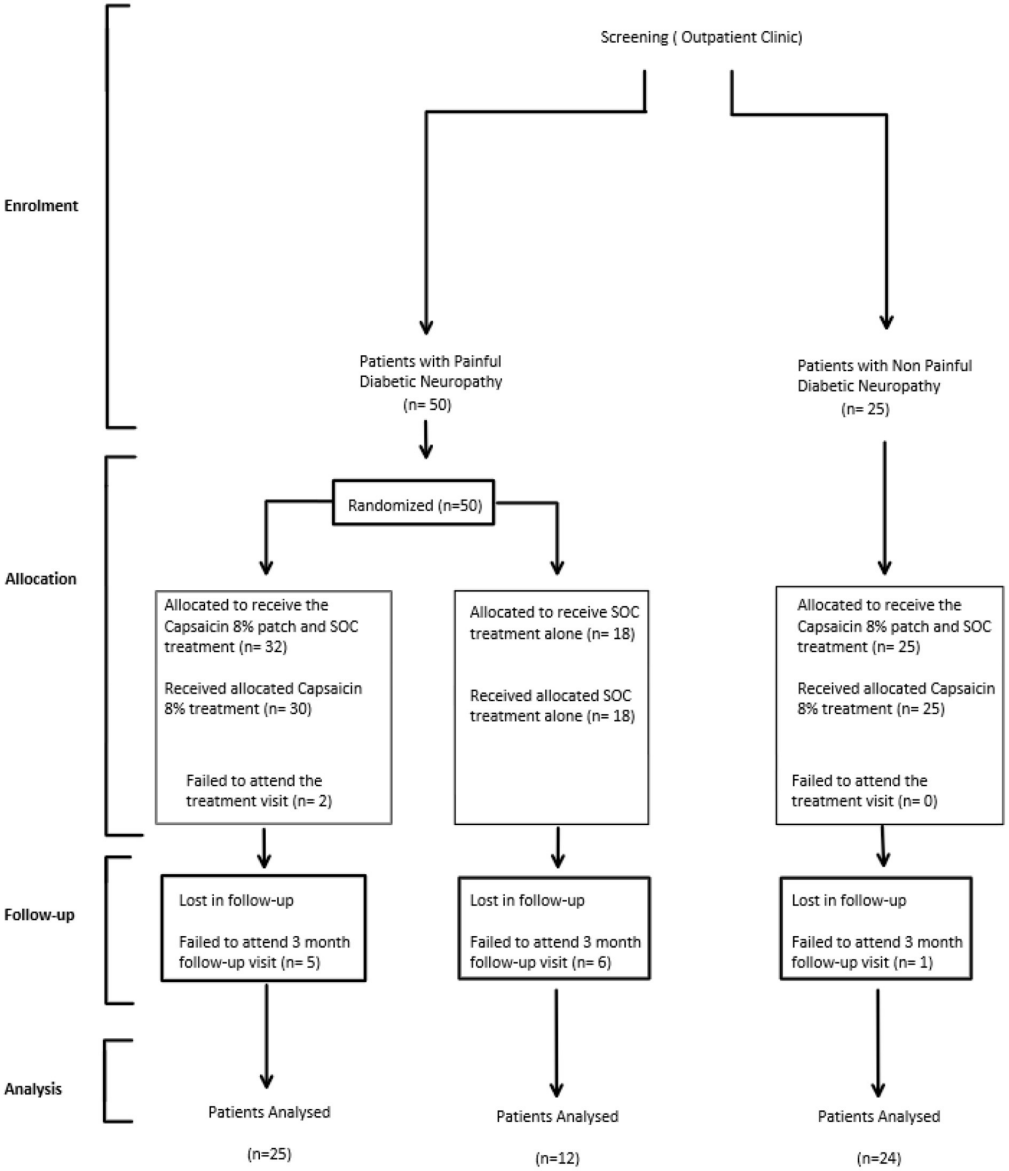
Participant flow diagram for DPN Capsaicin 8% patch studies.

Participants with Painful Diabetic Peripheral Neuropathy (PDPN) enrolled in the study were randomly allocated to either receive 30-min Capsaicin 8% patch application to both feet up to the distal calf, while continuing to take medication as part of their standard of care (PDPN Qutenza + SOC group; *n* = 32, *n* = 25 completers at 3 months; Figure 1), or to receive standard of care alone (PDPN SOC alone group; *n* = 18, *n* = 12 completers at 3 months Figure 1). Randomization was performed using a Sealed Envelope method and patches dispensed *via* Clinical Trial Pharmacist.

Participants with Non-Painful Diabetic Neuropathy enrolled in the study were not randomized, and they all received 30-min Capsaicin 8% patch application (*n* = 25, *n* = 24 completers at 3 months Figure 1).

**Figure 2:**
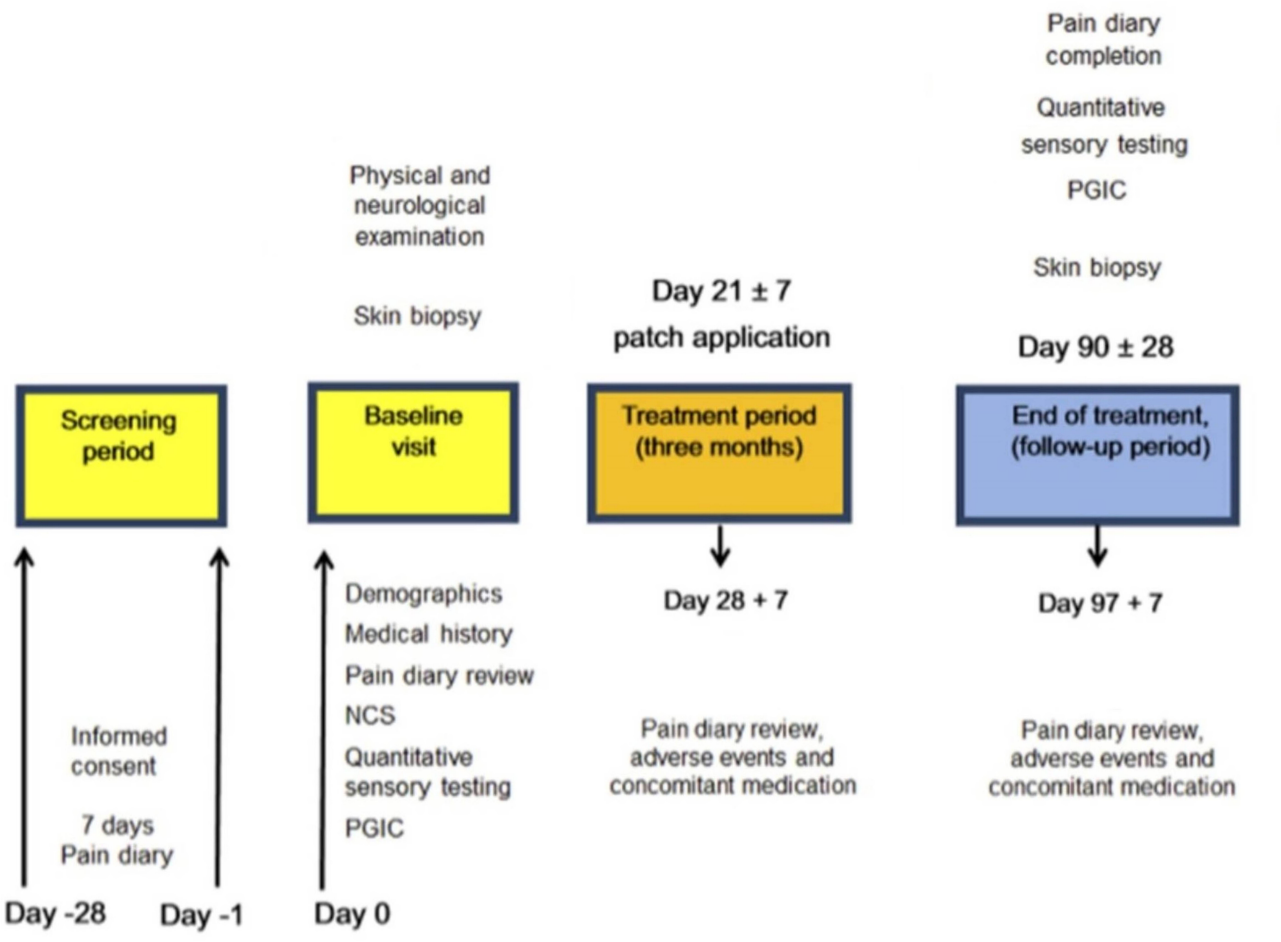
Study flow diagram illustrating the sequence of assessment for participants in the study. NCS, Nerve conduction study, PGIC, Patient Global Impression of Change.

The study involved hospital visits and telephone calls (Figure 2).

Participants had a follow-up visit at around 3 months from the treatment visit (Figure 2).

### Participants

Patient demographics for all patients are presented in Table 1. All patients had Diabetes of at least 1 year duration and distal neuropathy. Exclusion criteria included: Non-diabetic neuropathies; History of alcohol/substance abuse; Pregnancy or plan for pregnancy; Significant renal impairment; and Heart failure. For the patients with PDPN exclusion criteria also included any other painful medical conditions that may interfere with the assessment of change in neuropathic pain.

**Table 1:** Participant demographics.

**Group**			
**Number of patients**	**PDPN Q** + **SOC**	**PDPN SOC alone**	**NPDPN Q** + **SOC**
	***n** =* **25**	***n** =* **12**	***n** =* **24**
**Age, years**			
Mean ± SD	62 ± 11	64 ± 9.1	59 ± 13
Range	40–83	51–84	34–82
**Sex**, ***n (%)***			
Male	12 (48)	5 (41%)	17 (71%)
Female	13 (52%)	7 (59%)	7 (29%)
**Type of diabetes**, ***n*** **(%)**			
Type I	5 (20%)	1 (8%)	18 (75%)
Type II	20 (80%)	11 (92%)	6 (25%)
**Duration of diabetes, mean, years (range; SD)**			
	18 (1–44; 11.3)	15 (3–37; 11.1)	29 (5–58; 17)
**Medications for diabetes**, ***n*** **(%)**			
Insulin alone	6 (24%)	2 (16%)	14 (56%)
Metformin alone	7 (28%)	5 (42%)	1 (4%)
Insulin + Metformin	2 (8%)	0 (0%)	3 (12%)
Insulin or Metformin + thiazolidinediones / gliptins / (SGLT2) inhibitors / sulfonylureas / GLP agonist	10 (40%)	5 (42%)	6 (24%)
**HbA1c % mean (range), mmol/mol mean (range)**			
	7.3 % (5.6–9.0)	6.8 % (5.5–8.7)	8 % (6.0–9.0)
	56 mmol/mol (38–75)	51.5 mmol/mol (37–72)	62 mmol/mol (41–74)
**Time elapsed since diagnosis of DPN, mean; years (range; SD)**			
	7.3 (1–29; 6.8)	9.0 (3–27; 6.6)	7 (2–15; 4)
**NCS (mean** **±SEM; (range)**			
Peroneal motor action potential amplitude	3.6 ± 0.7	1.8 ± 0.7	2.0 ± 0.7
	(0.0–15.2) mV	(0.0–6.4) mV	(0.0–12.7) mV
Peroneal conduction velocity	38.7 ± 3.4	31.9 ± 5.8	19.1 ± 5.1
	(0.0–53) m/s	(0.0–55.6) m/s	(0.0 −54) m/s
Sural sensory action potential amplitude	6.9 ± 1.2	1.8 ± 0.6	3.7 ± 1.2
	(0.0–21.6) μV	(0.0–6.0) μV	(0.0–17) μV
Sural nerve conduction velocity	41.8 ± 4.9	27.0 ± 7.1	20 ± 5.2
	(0.0–70.2) m/s	(0.0–61.2) m/s	(0.0–54.5) m/s

All patients were given a trial diary to complete starting on the day of screening and continuing for the next 7 days. The diary collected pain rating scores twice daily (ranging from 0 to 10). An 11-point numerical rating scale (NPRS) with the 0-anchor point being “no pain” and the 10-anchor point being “pain as bad as you can imagine,” to describe “pain on average in the last 24 h” was used for both spontaneous and evoked pain.

After completing this diary for 7 days, the result of their NPRS was used to determine eligibility for the study.

Patients with PDPN were enrolled only when the average pain intensity was equal to or > 4 /10 for spontaneous pain. The participants were unaware of this criterion.

Patients with NPDPN were enrolled only if the pain score was equal to 0.

Patients with PDPN considered eligible were advised to continue completing the diary daily for the entire duration of the study until the end-of-study follow up visit. Capsaicin 8% patch treatment was within 1 month after the baseline visit.

### Pain characteristics

In the group of patients with PDPN, pain was described mostly as burning pain (64%) and less commonly as cramping pain (6%), sharp pain (11%), shooting pain (6%); throbbing/ stabbing pain (6%) or aching pain (3%).

29 out of 37 subjects were taking treatments for neuropathic pain at the start of the study (Pregabalin, Duloxetine, Amitriptyline, Gabapentin, NSAIDs, opioids, or a combination of these), but not topical treatments. The mean (SEM) of the numeric pain rating score (NPRS) at the first visit was 7.1 (0.31) for PDPN Q+SOC group and 8.1 (0.46) for PDPN SOC alone group. The mean ± SEM of the SF-McGill Questionnaire overall pain score at the first visit was 106 (9.5) for PDPN Q+SOC group and 126 (15.2) for PDPN SOC alone group. Pain characteristics for patients with PDPN are reported in Table 2.

**Table 2:** Pain characteristics of patients with diabetic neuropathy.

**Group**		
	**PDPN Q**+**SOC**	**PDPN SOC alone**
	***n** =* **25**	***n** =* **12**
**Time elapsed since onset of pain associated with PDPN, mean; years (range; SD)**		
	7.6 (1–24; 5.9)	8.5 (2–27; 6.9)
**Baseline pain score (NPRS) for spontaneous pain, mean (range; SD)**		
	7.1 (4.4–9.5; 1.6)	8.1 (4.8–10; 1.6)
**Medications for pain**, ***n (%)***		
Pregabalin	10 (40%)	8 (67%)
Duloxetine	6 (24%)	4 (33%)
Amitriptyline	4 (16%)	1 (8%)
Gabapentin	2 (8%)	1 (8%)
NSAIDs	5 (20%)	3 (25%)
Opioids	5 (20%)	6 (50%)

Patients with NPDPN did not report pain in the extremities; some described their symptoms as lack of sensation or numbness.

### Clinical examination

Clinical examination and tests were performed in both lower and upper limbs, and the right lower limb values were used for analyses.

Neurological deficits were recorded using the Neuropathy Impairment Score Lower Limbs (33) (NIS-LL) which is a summed score including of muscle power, reflex loss (maximum score 88, indicating severe neuropathy).

The mean ± SEM NIS-LL at the baseline visit was 10.7 (1.1) for PDPN Q+SOC group; 12 (0.8) PDPN SOC alone group and 9.6 (1.4) for patients with Non-Painful Diabetic neuropathy (NPDPN Q+SOC).

### Assessment of neuropathy

#### Short-form McGill pain questionnaire (SF-MPQ-2)

Symptoms were recorded using the Short-Form McGill Pain Questionnaire (34) with maximum score 220, indicating severe symptoms. Participants completed the SF-MPQ-2 by rating the extent to which they experienced each of 22 pain descriptors in the past week. The SF-MPQ-2 is composed of 4 summary scales: (1) continuous descriptors (throbbing pain, cramping pain, gnawing pain, aching pain, heavy pain, and tender); (2) intermittent descriptors (shooting pain, stabbing pain, sharp pain, splitting pain, electric-shock pain, and piercing); (3) neuropathic descriptors (hot-burning pain, cold freezing pain, pain caused by light touch, itching, tingling or pins and needles, and numbness); and (4) affective descriptors (tiring-exhausting, sickening, fearful, and punishing-cruel). A total pain score was computed by averaging participant ratings across all questions, while scale pain scores were derived from averaging ratings to questions that comprise the respective scales.

#### Patient global impression of change

Patients were asked to complete the Clinical Global Impression of Change; this was completed at the baseline visit (for over the previous 3 months) and at 3 months follow-up visit. The Questionnaire is composed of a 7-point scale, enabling the patient to indicate no change, improvement or worsening of their condition.

#### Nerve conduction studies

Nerve conduction studies were performed only on visit 1 by a senior consultant neurophysiologist. Nerve conduction studies of the common peroneal (including F wave studies) and sural nerves in the right leg were performed with a Medtronic Keypoint device (Medtronic, Minneapolis, MN, USA). Nerve conduction studies were performed once at the baseline visit for all patients. Sural antidromic sensory action potentials of <5 μV amplitude and <40 m/s conduction velocity, and common peroneal nerve (compound muscle action potential from extensor digitorum brevis) values <3 mV amplitude, <40 m/s conduction velocity, were considered abnormal (35).

#### Quantitative sensory testing

For quantitative sensory testing (QST), thresholds for light touch were measured using Semmes–Weinstein hairs (made by A. Ainsworth, University College London, UK); No. 1 (0.0174 g) to No. 20 (263.0 g). The hair with the lowest force reliably detected by the patient on the dorsum of the toe was recorded. Values > 0.0479 g were considered abnormal (35).

Vibration perception thresholds were measured using a biothesiometer (Biomedical Instrument Company, Newbury, OH, USA) placed on the metatarsophalangeal joint of the big toe. Three ascending and three descending trials were carried out, and the mean value obtained. Values >12 V were considered abnormal (36).

Thermal perception thresholds were performed as described (3, 37) using the TSA II–NeuroSensory Analyzer (Medoc, Ramat Yishai, Israel). A 30 mm × 30 mm thermode was used and thermal thresholds determined in the soles of the feet (under the instep), for warm perception, cool perception, heat pain and cold pain from a baseline temperature of 32°C, with a change in temperature of 1°C /s. The mean of three consecutive tests for each modality was recorded. Values >6.4°C for warm sensation, >2.3°C for cool sensation and >10.4°C for heat pain, were considered abnormal (3, 35, 37).

#### Axon-reflex vasodilatation

Axon reflex vasodilatation was assessed after the topical application of 2 cm^2^ capsaicin 8% patch on the dorsum foot skin for 10 min. The increase of capillary flux (peak minus baseline) was measured adjacently using a laser-Doppler (Perimed, Stockholm), and recorded in flux units (3). This was assessed only in a subset of participants, for reasons related to availability of equipment.

#### Calf skin biopsy and immunohistochemistry

3.5-mm diameter skin punch biopsies were collected under local anesthesia from the distal lateral calf of patients at baseline (visit 1) before capsaicin 8% patch (Qutenza) application and repeated 3 months after the initial visit or treatment, within the area of capsaicin 8% patch treatment. Skin biopsies collected from age and gender-matched healthy volunteers were analyzed alongside the patient‘s biopsies, as controls.

The immunohistochemical methods and antibodies used have been reported previously (17, 22, 38–40). One of the two skin biopsies was snap frozen and stored at −70°C, and the other immersed in fixative (modified Zamboni's fluid-−2% formalin; 0.01 M phosphate buffer; 15% saturated picric acid (pH 7.2), then washed in phosphate-buffered saline (PBS; 0.1 M phosphate; 0.9% w/v saline; pH 7.3) containing 15% w/v sucrose for an hour, before snap freezing in optimum cutting tissue embedding medium (Tissue-Tek OCT, RA Lamb Ltd, Eastbourne, U.K.). Frozen sections (15 μm thickness) were collected onto poly-L-lysine (Sigma, Poole, UK) coated glass slides and post-fixed in freshly prepared, 4% w/v paraformaldehyde in 0.15 M phosphate-buffered saline (PBS) for 30 min. Sections of pre-fixed tissue were collected in the same way and allowed to air dry for markers. Endogenous peroxidase was blocked by incubation in methanol containing 0.3% w/v hydrogen peroxide for 30 min for both post- and pre-fixed sections. After rehydration, appropriately processed sections were incubated overnight with primary antibodies.

The primary antibodies were to the structural nerve marker PGP 9.5 (Rabbit, RA95/06, 1:40,000; Ultraclone, Isle of Wight, UK), the nerve regeneration marker, growth associated protein GAP-43 (G9264, Mouse, 7B10, 1:80,000; Sigma, Poole, UK), von Willebrand factor vWF (Rabbit, 1:10,000; Novocastra Laboratories, Milton Keynes, UK). 50-μm sections were also studied with PGP 9.5 antibody.

Briefly, fixed sections were floated onto PBS in 12-well plates, dehydrated with alcohol/hydrogen peroxide solution for 30 min, washed with PBS and incubated with PGP 9.5 overnight, washed and incubated with the second antibody for 1 h, and then washed and incubated with ABC as above. After washing, the nickel developer solution was added, and staining allowed to develop. The reaction was stopped by adding 0.1 M sodium acetate pH 6.0, washed again in PBS, counterstained and free floated onto PPL slides, allowed to dry and incubated in xylene, and finally mounted using DPX mountant.

Nerve fibers were counted along the length of four non-consecutive sections. The length of epithelium in each counted section was measured using computerized microscopy software (Olympus ANALYSIS 5.0 Soft, Olympus UK, Southend, Essex, UK) and results expressed as fibers/mm length of the section. Sub-epidermal nerve immunoreactivity was obtained as a percentage (% area) measured by image analysis of digital photomicrographs captured *via* video link to an Olympus BX50 microscope. The gray-shade detection threshold was set at a constant level to allow detection of positive immuno-staining and the area of highlighted immuno-reactivity was expressed as a percentage (% area) of the field scanned. Images were captured (x40 objective magnification) along the entire length, and the mean values were used for statistical analysis. Quantification was performed by two independent blinded observers, and there was no significant difference between observers. IENF density for PGP 9.5 in 50 μm thickness sections were quantified according to the European Federation of Neurological Societies/Peripheral Nerve Society Guidelines (41). Validation of these methods, including vs. PGP9.5 IENF in 50 μm vs. 15 μm thickness sections, have been published previously (42, 43). The GAP 43 IENFs were sparse and so only SENFs were analyzed [as in Narayanaswamy et al. (24)].

#### Statistical analysis

Data were analyzed using GraphPad Prism version 5.0 for Windows (GraphPad Prism Software, San Diego, CA, USA). The statistical tests used were paired two-tailed Mann-Whitney test, Student's *t*-test, two-way ANOVA analysis, and Spearman's correlation test. For all statistical tests, *p***-**values < 0.05 were considered significant.

## Results

Data are presented for participants who completed all tests at the 3-month follow up visit (PDPN Q+SOC, *n* = 25; PDPN SOC alone, *n* = 12; NPDPN Q+SOC, *n* = 24). Few participants had further visits, mainly on account of the Covid-19 pandemic (data not shown). The control of diabetes was not significantly different at baseline across the study groups, or after patch treatment—in the PDPN Q+SOC group, pre-treatment Mean [range] HbA1c (mmol/mol), 55.0 (38–75), post-treatment 59.5 (42–90), *p* = 0.93; in the NPDPN Q+SOC group, pre-treatment 60 (41–77), and post-treatment 57 (45–69), *p* = 0.31.

### Clinical examination and assessment of neuropathy

Clinical examination and tests confirmed that patients had a sensory, length-dependent neuropathy.

Patients with Painful Diabetic Neuropathy (PDPN Q+SOC; *n* = 25; *p* = 0.63) did not show significant difference in the NIS-LL score before and after the treatment of Capsaicin 8% patch.

Similarly, no statistically significant changes in NIS-LL were observed in the group of patients with Painful Diabetic Neuropathy who did not receive the treatment with capsaicin 8% patch (PDPN SOC alone; *n* = 12; *p* = 0.88) and for patients with non-Painful Diabetic Neuropathy (NPDPN Q+SOC; *n* = 24; *p* = 0.14; Table 3).

**Table 3:** Skin biopsy markers assessed at baseline and at 3 months follow–up visit in PDPN Q+SOC and PDPN SOC alone groups, for PGP 9.5 in 15μm and 50μm thick sections.

			**PDPN Q+ SOC (*n* = 25)**		**PDPN SOC alone (*n* = 12)**		**NPDPN Q** +**SOC (*n* = 24)**	
**PGP IENFs**	**15μm**		**Mean ±SEM**	***P*-value**	**Mean ±SEM**	***P*–value**	**Mean ±SEM**	***P*-value**
		Baseline	0.70 ± 0.23	^***^p = 0.0005	0.02 ± 0.03	*p =* 0.62	0.64 ± 0.17	^*^p = 0.02
		3 months FU	1.72 ± 0.33		0.05 ± 0.06		1.38 ± 0.44	
	50μm	Baseline	1.50 ± 0.45	^***^p = 0.0002	0.15 ± 0.10	*p =* 0.57	1.27 ± 0.35	^**^p = 0.002
		3 months FU	3.46 ± 0.67		0.24 ± 0.20		3.0 ± 0.79	
**PGP SENFs**	15μm	Baseline	0.55 ± 0.06	^**^p = 0.003	0.24 ± 0.04	*p =* 0.95	0.25 ± 0.04	^***^p < 0.0001
		3 months FU	0.79 ± 0.07		0.23 ± 0.04		0.48 ± 0.08	
	50μm	Baseline	1.06 ± 0.11	^***^p < 0.0001	0.43 ± 0.05	*p =* 0.85	0.68 ± 0.11	^***^p = 0.0004
		3 months FU	1.62 ± 0.18		0.44 ± 0.07		1.15 ± 0.18	
**GAP SENFs**		Baseline	0.26 ± 0.05	^**^p = 0.003	0.11 ± 0.03	*p = 0.04	0.33 ± 0.07	^***^p = 0.0005
		3 months FU	0.46 ± 0.07		0.06 ± 0.02		0.60 ± 0.09	
**Vwf**		Baseline	4.2 ± 0.23	*p =* 0.18	5.15 ± 0.47	*p =* 0.7	4.20 ± 0.20	*p =* 0.38
		3 months FU	4.5 ± 0.38		5.30 ± 0.37		4.32 ± 0.20	

IENFs, Intra–epidermal Nerve Fiber density (fibers/mm); SENFs (% Area), Sub–epidermal Nerve Fiber density; PGP 9.5, Pan–neuronal marker protein gene product 9.5; GAP 43, Growth–associated protein 43; vWF, von Willebrand Factor.

* Significant,

** Very significant,

*** Highly significant.

### Nerve conduction studies

Results for the nerve conduction studies are reported in Table 1.

Most patients (78%; 48/61) had at least one abnormality on nerve conduction studies; 54% (33/61) had both motor and sensory abnormalities. The peroneal conduction motor response was absent in 4 patients in the PDPN Q+SOC group, 4 patients in the PDPN SOC alone group and 11 patients in the NPDPN Q+SOC group. The sural nerve response was absent in 6 patients in PDPN Q+SOC group, 5 patients in PDPN SOC alone group and 14 patients in the NPDPN Q+SOC group.

### Pain scores and questionnaires

**Figure 3:**
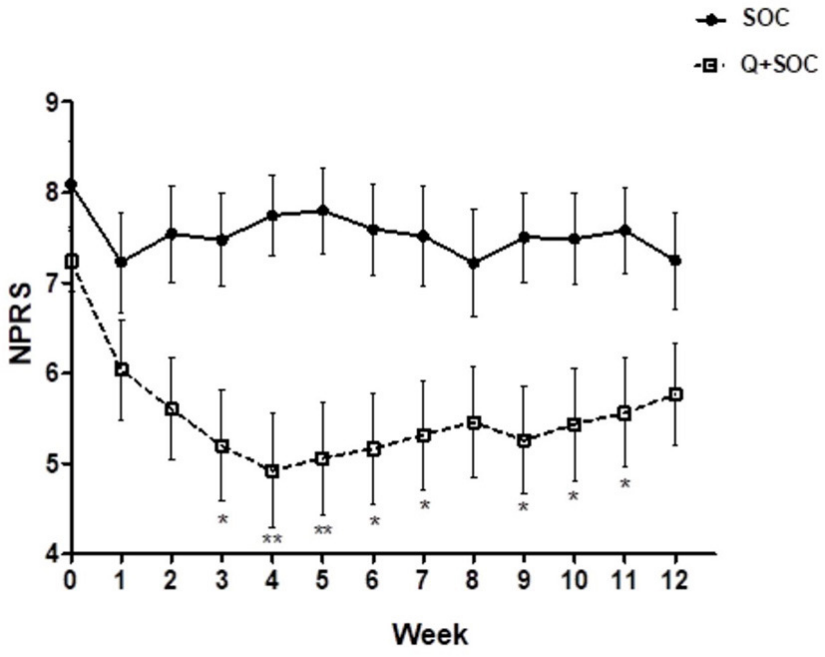
Time–course graph of spontaneous pain scores over 12 weeks in the two study groups, capsaicin 8% patch Qutenza (Q) plus Standard of Care group (PDPN Q+SOC; *n* = 25), and Standard of Care group (PDPN SOC alone; *n* = 12). Separate assessment of pain scores at each week time point showed a significant difference in NPRS scores at week 3 (*p < 0.05), week 4 (**p < 0.05), week 5 (*p < 0.05), week 6 (*p < 0.05) and week 9 (*p < 0.05; two–way ANOVA analysis).

**Figure 4:**
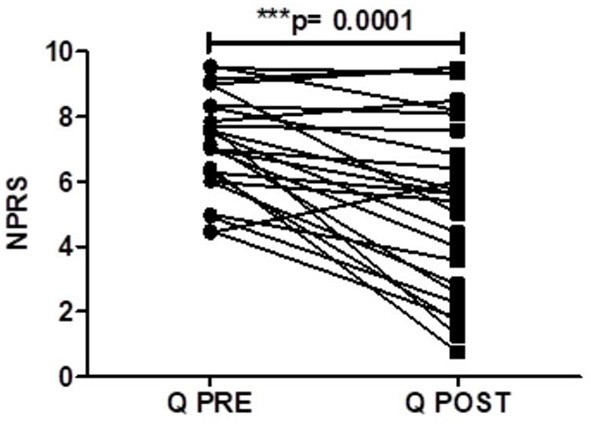
Pain scores of patients (PDPN Q + SOC, *n* = 25) treated with capsaicin 8% patch Qutenza (Q), at pre–treatment visit (Q PRE) and 3 months after treatment (Q POST), on Numerical Pain Rating Scale (NPRS). Statistically significant difference at 3 months after treatment (***p = 0.0001; *n* = 25; paired *t*–test).

There was a significant reduction in the average daily NPRS 3 months after capsaicin 8% patch application for the PDPN Q+SOC group (Figure 3), with a mean difference of NPRS between baseline and 3-month follow-up visit −1.87, ^***^p = 0.0001 (Figure 4). The change in average pain score at the 3-month follow-up visit for the SOC alone group was not significant (mean difference of NPRS between baseline and 3 months follow-up −0.58, *p* = 0.11).

At the follow-up visit 3 months after the treatment for PDPN Q+SOC group, SF-MPQ-2 showed a significant reduction in the overall pain score (−31.1; ^**^p = 0.002), intermittent descriptors (−9.4; ^**^p = 0.005), neuropathic descriptors (−5.9; ^*^p = 0.02) and affective descriptors (−9.4; ^**^p = 0.001). There was no significant difference in these scores in the PDPN SOC alone group.

Patient with non-painful Diabetic Neuropathy (NPDPN Q+SOC) did not report pain in the feet or distal calf for the entire duration of the study.

Patient's Global Impression of Change (PGIC) was not significantly improved in the PDPN Q+SOC group (PGIC score at baseline: 4.0 ± 0.0; PGIC score at 3 months follow-up visit: 3.0 ± 0.3; ^**^p = 0.007), or in the PDPN SOC alone group (PGIC score at baseline: 3.7 ± 0.2; PGIC score at 3 months follow-up visit: 4.0 ± 0.2; *p* = 0.11).

The PGIC in patents with non-painful Diabetic Neuropathy (NPDPN Q+SOC) was recorded as improvement of sensation at 3 months after the treatment (^**^p = 0.002).

### Quantitative sensory testing

**Figure 5:**
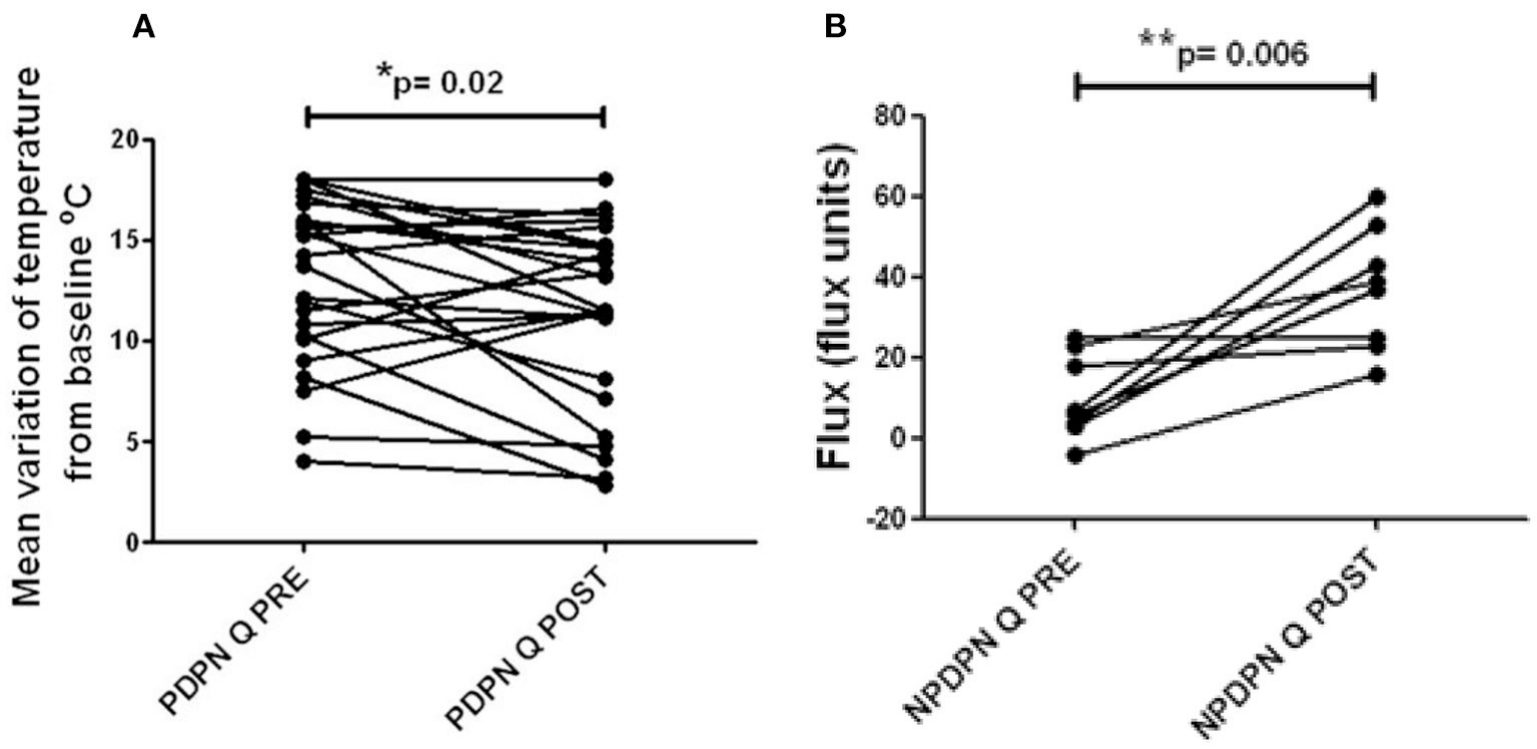
Warm thermal thresholds in patients with DPN. **(A)** Thermal thresholds for warm perception (changes from baseline at 32°C), in PDPN Q + SOC group treated with capsaicin 8% patch Qutenza (Q), at pre–treatment visit (Q PRE) and visit 3 months after treatment (Q POST). Statistically significant difference (improvement) 3 months after the treatment (*p = 0.02; paired *t*–test). **(B)** Axon reflex vasodilatation flux in patients with NPDPN group treated with capsaicin 8% patch Qutenza (Q) at pre–treatment visit (Q PRE) and visit 3 months after treatment (Q POST). Statistically significant difference (improvement) 3 months after the treatment (*p = 0.006; paired *t*–test).

There were no significant changes in the touch, vibration, cool or heat pain thresholds, in PDPN Q+SOC, PDPN SOC alone and NPDPN Q+SOC groups. The PDPN Q+SOC group showed a statistically significant improvement of warm perception threshold (mean ± SEM (range) from baseline 13.1 (0.8) at 3 months after Capsaicin 8% patch application (mean ± SEM (range) 11.4 (0.9); ^*^p = 0.02; Figure 5A). There was no change in thresholds in the PDPN SOC alone group. Patients with Non-painful Diabetic Neuropathy (NPDPN Q+SOC) showed a trend toward an improvement of the warm threshold, but it was not statistically significant.

### Axon-reflex vasodilatation

Axon-reflex vasodilatation was assessed in a subset of participants with non-painful diabetic neuropathy (NPDPN Q+SOC). Axon-reflex vasodilatation in skin was significantly increased between baseline and 3-month follow-up visit (*n* = 7, ^**^p = 0.006, Figure 5B). In a previous study, capsaicin-induced axon-reflex vasodilatation in dorsum foot skin was approximately 50% lower in DPN than in matched healthy volunteers (3).

### Immunohistochemistry

#### PGP9.5 IENFs

**Figure 6:**
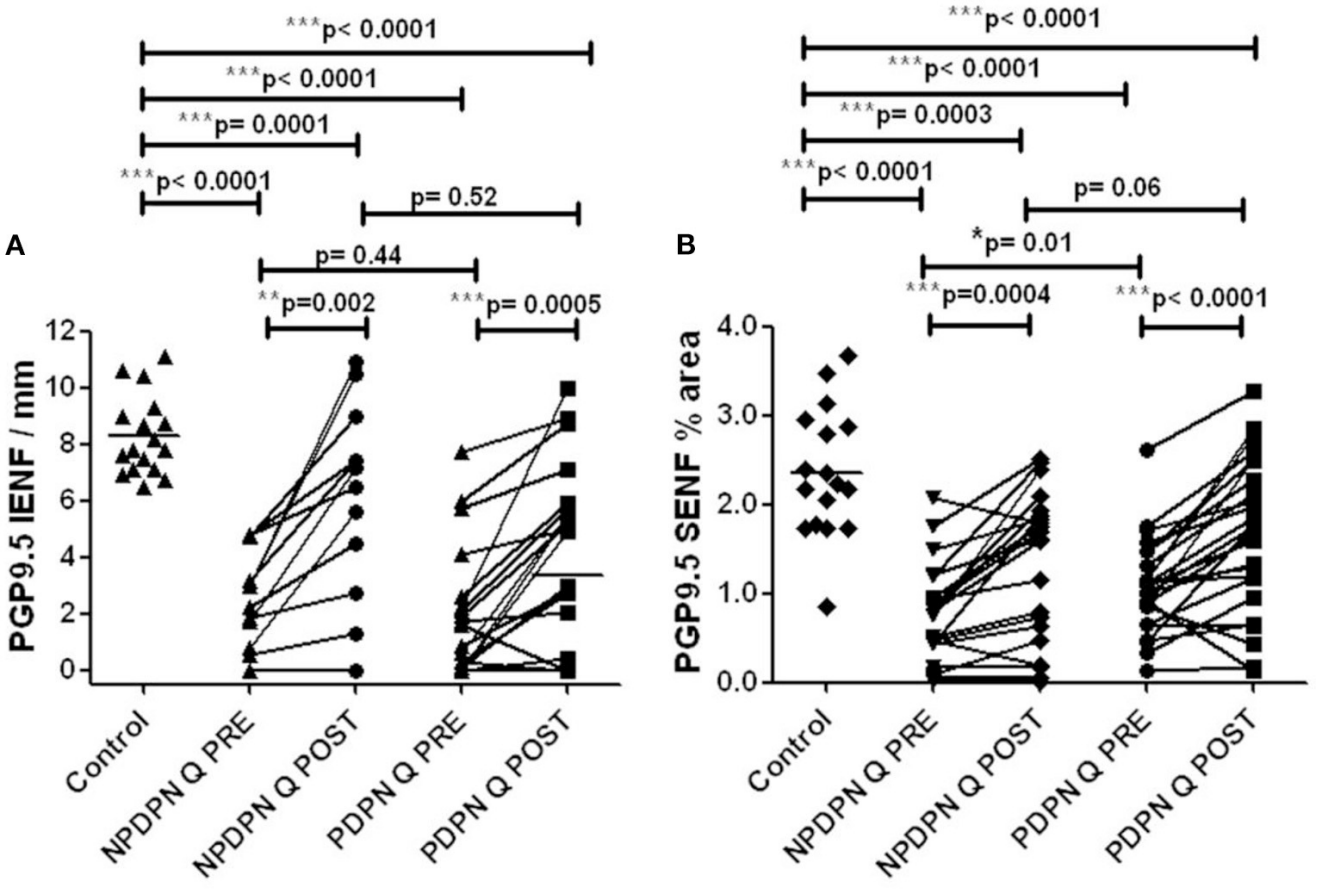
PGP9.5 immunohistochemistry in 50 μm sections from skin biopsies in non–painful (NPDPN Q+SOC) and painful (PDPN Q+SOC) Diabetic Peripheral Neuropathy following Capsaicin 8% patch Qutenza (Q) treatment. Intra–epidermal (IENF/mm, A) and sub–epidermal fiber density (SENF, % area, B) in NPDPN and PDPN. **(A)** IENFs in NPDPN Q +SOC and PDPN Q+SOC at baseline (Q PRE) and statistically non–significant difference before (Q PRE, *p* = 0. 44; Mann–Whitney test; *n* = 24 NPDPN Q+SOC and *n* = 25 PDPN Q+SOC) or 3 months after treatment (Q POST, *p* = 0.52, Mann–Whitney test). Statistically significant difference between control group and baseline (Q PRE), and after treatment (Q POST) (Mann–Whitney test). **(B)** SENFs in NPDPN Q+SOC and PDPN Q+SOC at baseline (Q PRE) and statistically significant difference before (Q PRE, *p = 0. 01; Mann–Whitney test; *n* = 24 NPDPN Q+SOC and *n* = 25 PDPN Q+SOC) but not 3 months after treatment (Q POST, *p* = 0.06, Mann–Whitney test). Statistically significant decreases between control group and baseline (Q PRE) but less significance after treatment (Q POST) (Mann–Whitney test) in NPDPN Q+SOC but not PDPN Q+SOC. ***Highly significant.

**Figure 7:**
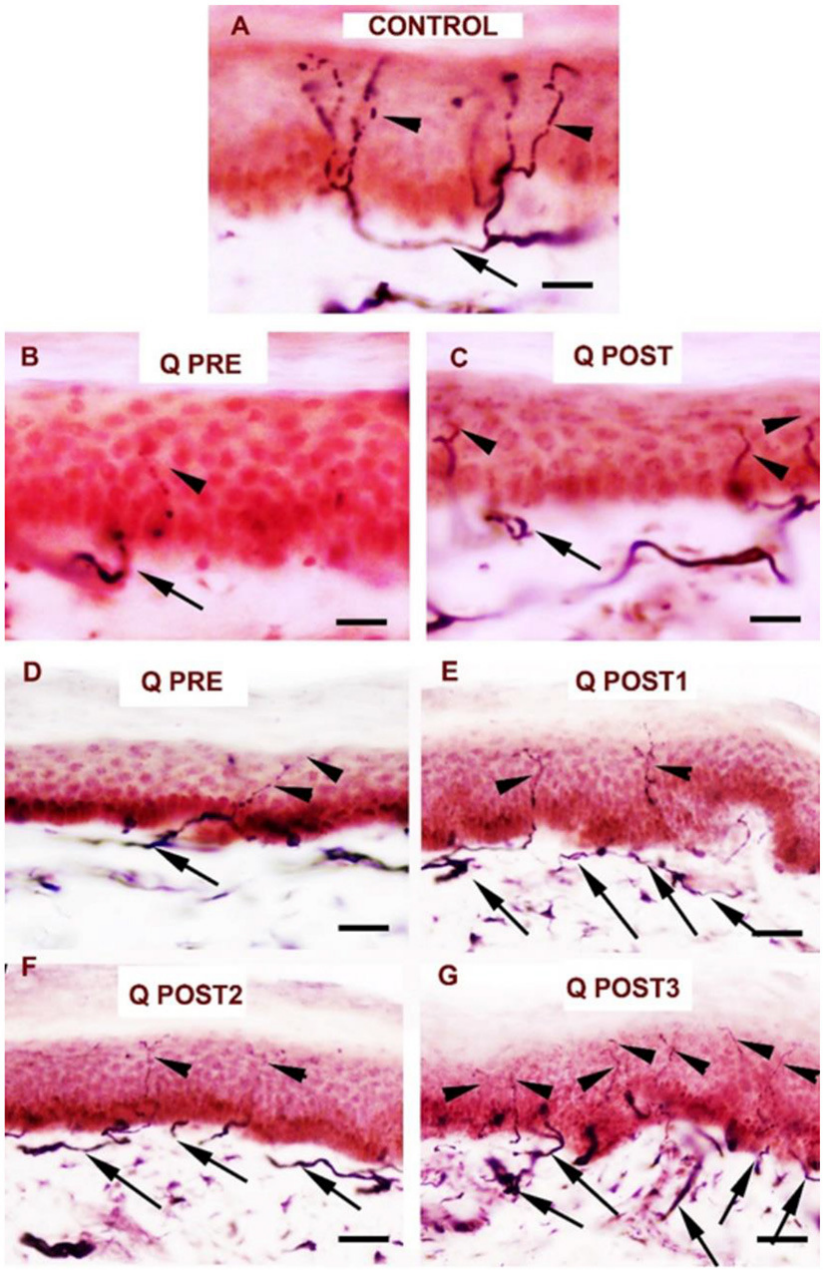
Staining for PGP9.5 in control skin, NPDPN Q +SOC and PDPN Q+SOC before and after application of Capsaicin 8% patch Qutenza (Q) treatment (50 μm sections). **(A)** Control skin biopsy section, from a healthy human volunteer (intra–epidermal nerve fibers marked with arrowheads and sub–epidermal nerve fibers with arrows). **(B)** Skin biopsy section from a subject with NPDPN Q+SOC pre–treatment (Q PRE); few intra–epidermal nerve fibers and sub–epidermal nerve fibers were observed before treatment. **(C)** Skin biopsy section from same subject with painless DPN (NPDPN Q+SOC) as above post–treatment (Q POST): the abundance of both the IENFs and SENFs appeared restored. (Scale bar 50 μm, Original magnification x40). **(D)** Skin biopsy section from a subject with PDPN Q+SOC pre–treatment (Q PRE); Few intra–epidermal nerve fibers and with abnormal trajectory before capsaicin application (arrowheads). **(E–G)** Examples from biopsies collected 3. 6 and 9 months (Q POST 1, 2, 3) after a single Qutenza application respectively. Note the restored abundance of both IENF and SENF, and the vertical trajectory of IENF Q POST, as observed in control skin. (Scale bar 100 μm, original magnification x20).

Calf skin biopsies collected at baseline and 3 months after the treatment with Capsaicin 8% patch showed, in the PDPN Q+SOC group (*n* = 25), a statistically significant increase of IENFs with PGP9.5 in 15 μm sections (^***^p = 0.0005) and 50-μm sections (^***^p = 0.0002, Figures 6A,B, 7).

No statistically significant changes were observed in the PDPN SOC alone group (*n* = 12) between baseline and biopsies after 3 months (Table 3).

Skin biopsies collected before and after the treatment with capsaicin 8% patch showed, in the NPDPN Q+SOC group (*n* = 24), a statistically significant increase of PGP9.5 marker for IENFs in 15 μm sections (^*^p = 0.02) and 50-μm sections (^**^p = 0.002, Figures 6A,B, 7).

#### PGP9.5 SENFs

At 3 months after treatment, PDPN Q+SOC group showed a statistically significant increase of PGP9.5 SENFs in 15 μm sections (^**^p = 0.003), and 50-μm calf skin sections (^***^p = 0.0001; Figures 6A,B, 7). PGP9.5 IENFs, before and after capsaicin 8% patch application, showed statistically significant differences between controls and the PDPN Q+SOC group, before but not after capsaicin 8% patch application, for both 15 μm and 50 μm thick sections. The NPDPN Q+SOC group showed a statistically significant increase of PGP9.5 SENFs in 15 μm sections (^***^p < 0.0001), and 50-μm calf skin sections (^***^p = 0.0004; Figures 6A,B, 7).

No statistically significant changes were observed in the PDPN SOC alone group (Table 3).

#### GAP43

**Figure 8:**
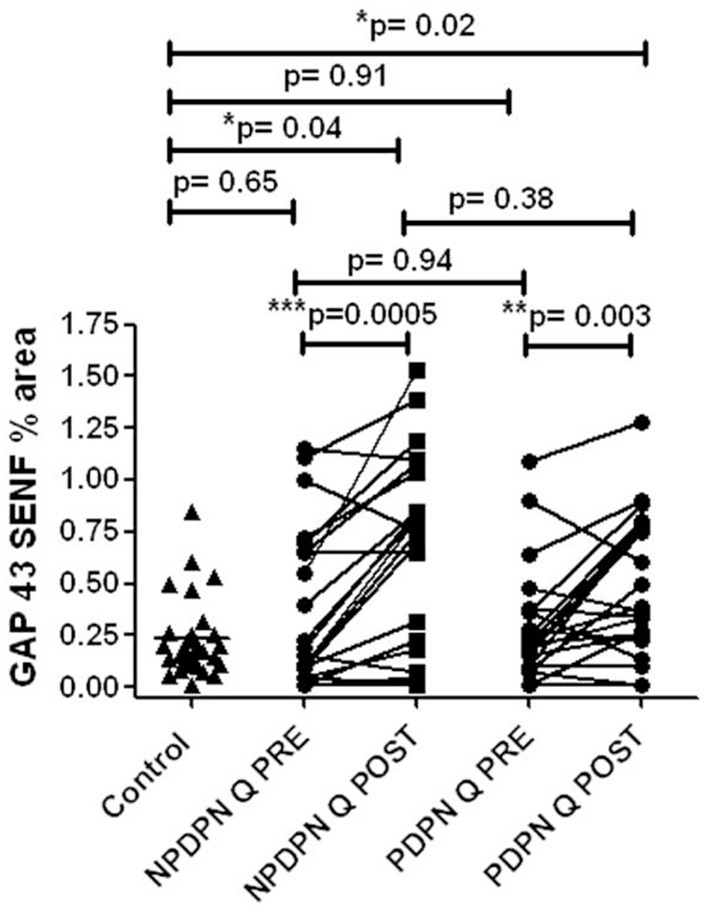
GAP 43 immunohistochemistry from skin biopsies in non–painful (NPDPN Q+SOC) and painful (PDPN Q+SOC) Diabetic Peripheral Neuropathy following Capsaicin 8% patch Qutenza (Q) treatment. Sub–epidermal fiber density (SENF, % area) in 30 μm sections in NPDPN Q+SOC and PDPN Q+SOC at baseline (Q PRE) and statistically no significant difference in NPDPN and PDPN before (Q PRE, *p* = 0. 94; Mann–Whitney test; *n* = 24 NPDPN Q+SOC) or 3 months after treatment (Q POST, *p* = 0. 38, Mann–Whitney test). Statistically no significant difference between control group and baselines (Q PREs), but significant increases after treatment (Q POSTs) in both NPDPN Q+SOC and PDPN Q+SOC (*p = 0.04 and *p = 0.02 respectively, Mann–Whitney test). **Very significant, ***Highly significant.

**Figure 9:**
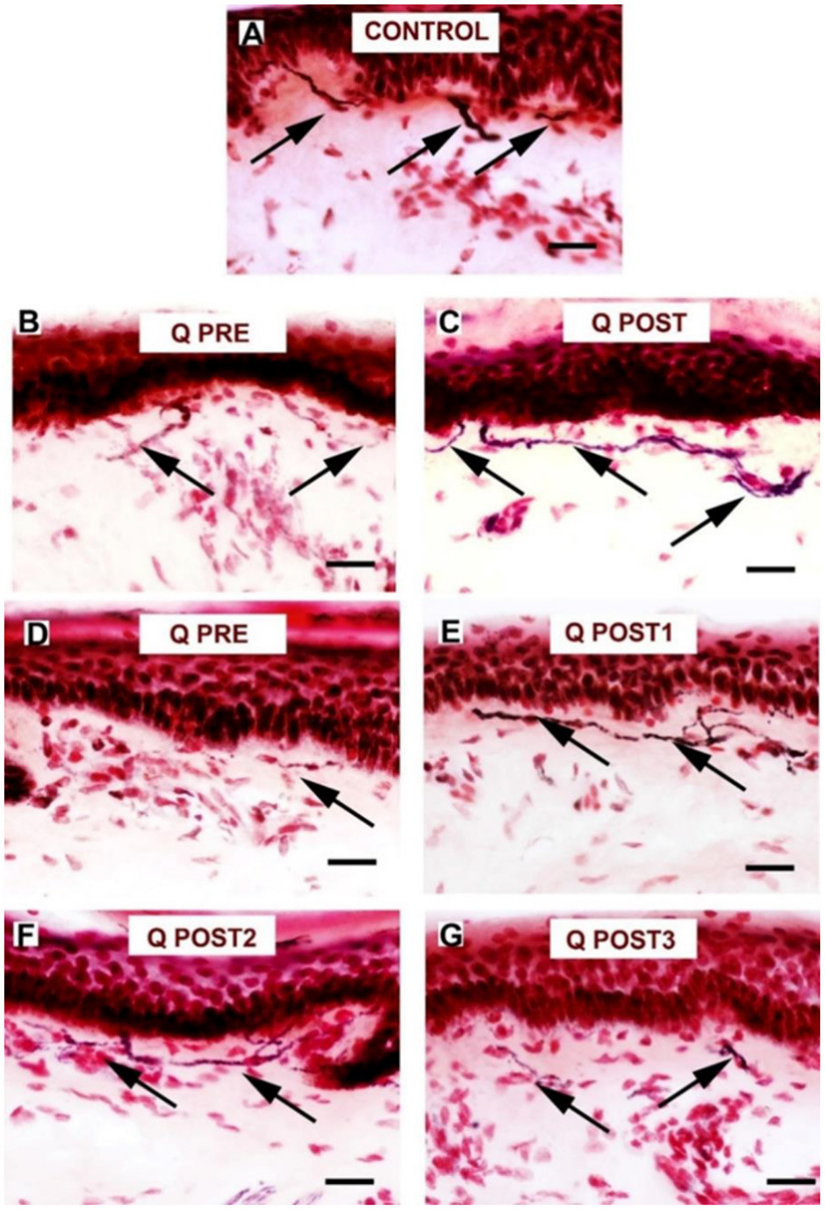
Staining for GAP 43 in control skin, NPDPN Q+SOC and PDPN Q+SOC before and after application of Capsaicin 8% patch Qutenza (Q) **(**treatment Control skin biopsy section, from a healthy human volunteer (sub–epidermal nerve fiber with arrow). **(A)** Control skin biopsy section, from a healthy human volunteer (sub–epidermal nerve fibers marked with arrows). **(B)**Skin biopsy section from a participant with NPDPN Q+SOC pre–treatment (Q PRE). **(C)** Same participant as above 3 months post–treatment (Q POST); Note the abundance and length of SENFs which appeared increased. **(D)** A participant with PDPN Q+SOC pre–treatment before treatment (Q PRE). **(E)** Same participant as above 3 months post–treatment (Q POST1); Note the abundance and length of SENFs which appeared increased. **(F,G)** Same PDPN Q+SOC participant 6 months post treatment (Q POST2) and 9 months post treatment (Q POST3), note return to control level at 9 months. (Scale bar 50 μm, Magnification x40).

The PDPN Q+SOC group showed a significant increase of GAP43 for SENFs compared to baseline at 3 months after treatment (^**^p = 0.003; Figures 8A,B, 9). Comparison between controls and PDPN Q+SOC group for GAP43 SENFs showed no significant difference before capsaicin 8% patch application, but these were significantly higher at 3 months after capsaicin 8% patch treatment in the PDPN Q+SOC group (^*^p = 0.02**)**. In contrast, a significant decrease was observed over 3 months in the PDPN SOC alone group; ^*^p = 0.04 (Table 3).

In skin biopsies collected 3 months after the treatment with capsaicin 8% patch, the NPDPN Q+SOC group showed a significant increase of GAP43 positive SENFs compared to baseline biopsies (^***^p = 0.0005; Figures 8A,B, 9).

Comparison between control and patient groups for GAP43 SENFs showed significant differences after capsaicin 8% patch application, but not before the treatment (Figure 8).

#### von Willebrand Factor (vWF)

**Figure 10:**
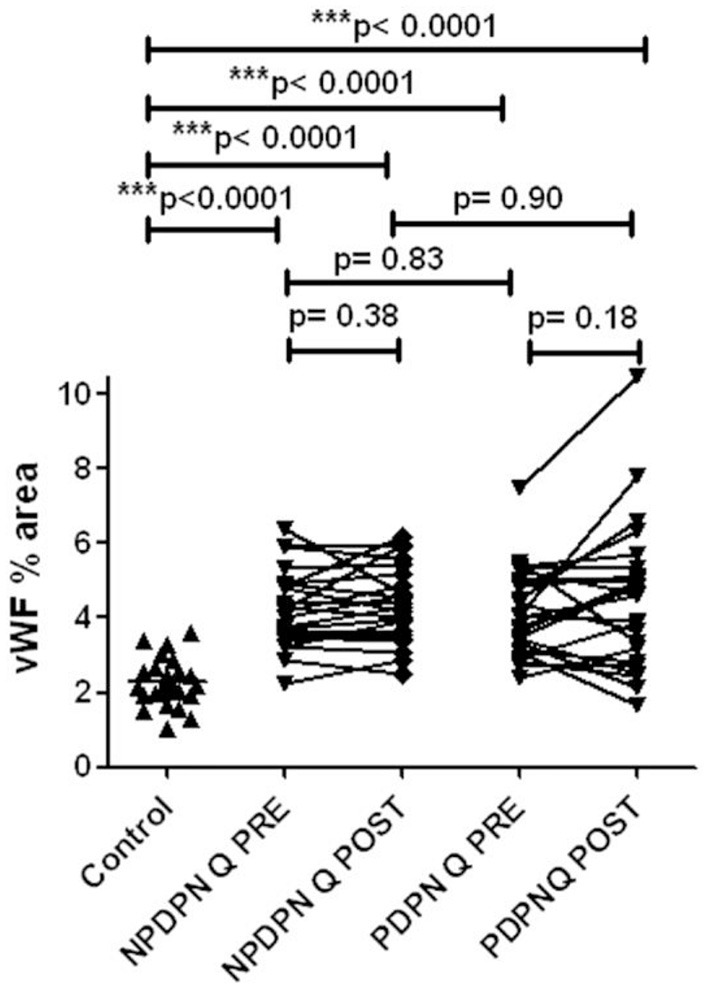
vWF immunohistochemistry in skin biopsies in non–painful (NPDPN Q+SOC) and painful (PDPN Q+SOC) Diabetic Peripheral Neuropathy following Capsaicin 8% patch Qutenza (Q) treatment. Vessel density (vWF, % area) in NPDPN Q+SOC and PDPN Q+SOC at baseline (Q PRE) and statistically no significant differences between NPDPN and PDPN before (Q PRE, *p* = 0. 83; Mann–Whitney test; *n* = 24 NPDPN Q+SOC) or 3 months after treatment (Q POST, *p* = 0. 90, Mann–Whitney test). Statistically significant increases (all ***p < 0.0001, Mann–Whitney test) between control group and baselines (Q PRE) or 3 months after treatment (Q POST) in both NPDPN Q+SOC and PDPN Q+SOC.

**Figure 11:**
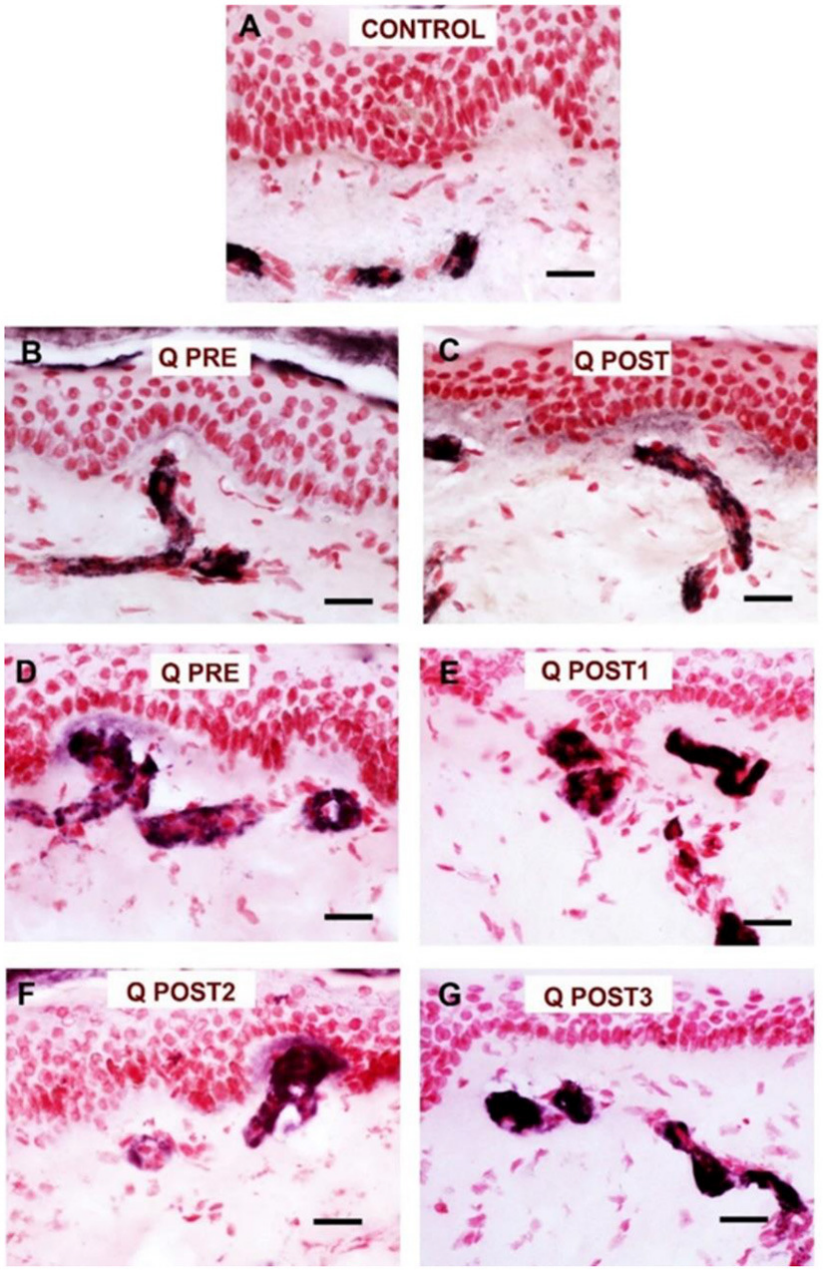
Staining for vWF in NPDPN Q+SOC and PDPN Q+SOC after of Capsaicin 8% patch Qutenza (Q) treatment. **(A)** Control skin biopsy from a human healthy volunteer (vessels stained in black). **(B)** NPDPN Q+SOC: Skin biopsy from subject pre–treatment (Q PRE); note increase in vessels compared to control. **(C)** Skin biopsy section from same NPDPN Q+SOC subject 3 months post–treatment (Q POST, blood vessels remain unchanged. **(D)** Skin biopsy from subject with PDPN Q+SOC pre–treatment (Q PRE); note increase in vessels compared to control and similar to NPDPN Q+SOC. **(E)** Skin biopsy section from same PDPN Q+SOC subject at 3 months post–treatment (Q POST1); blood vessels remain elevated and unchanged. **(F,G)** Further follow–ups in PDPN Q+SOC at 6 and 9 months (Q POST2 and 3, respectively) after treatment. (Scale bar 50 μm, Magnification x40).

No changes in von Willebrand Factor (vWF) were observed in the PDPN Q+SOC group after treatment (Figures 10, 11). No significant changes were observed in the PDPN SOC alone group (Table 3).

No changes in von Willebrand Factor (vWF) were observed after treatment with capsaicin 8% patch in patients with non-Painful Diabetic Neuropathy (NPDPN Q+SOC; Figures 10, 11).

Comparison between controls and patients with diabetic neuropathy before and after capsaicin 8% patch application showed significantly higher levels of vWF (Figure 10) in DPN, as we have reported previously in a different cohort of PDPN/NPDPN patients (6).

### Responder analyses

Thermal thresholds for warm perception in the PDPN Q + SOC group were significantly improved 3 months after capsaicin 8% patch treatment and were positively correlated with the improvement in the overall pain score (SF-MPQ, ^*^p = 0.04).

**Figure 12:**
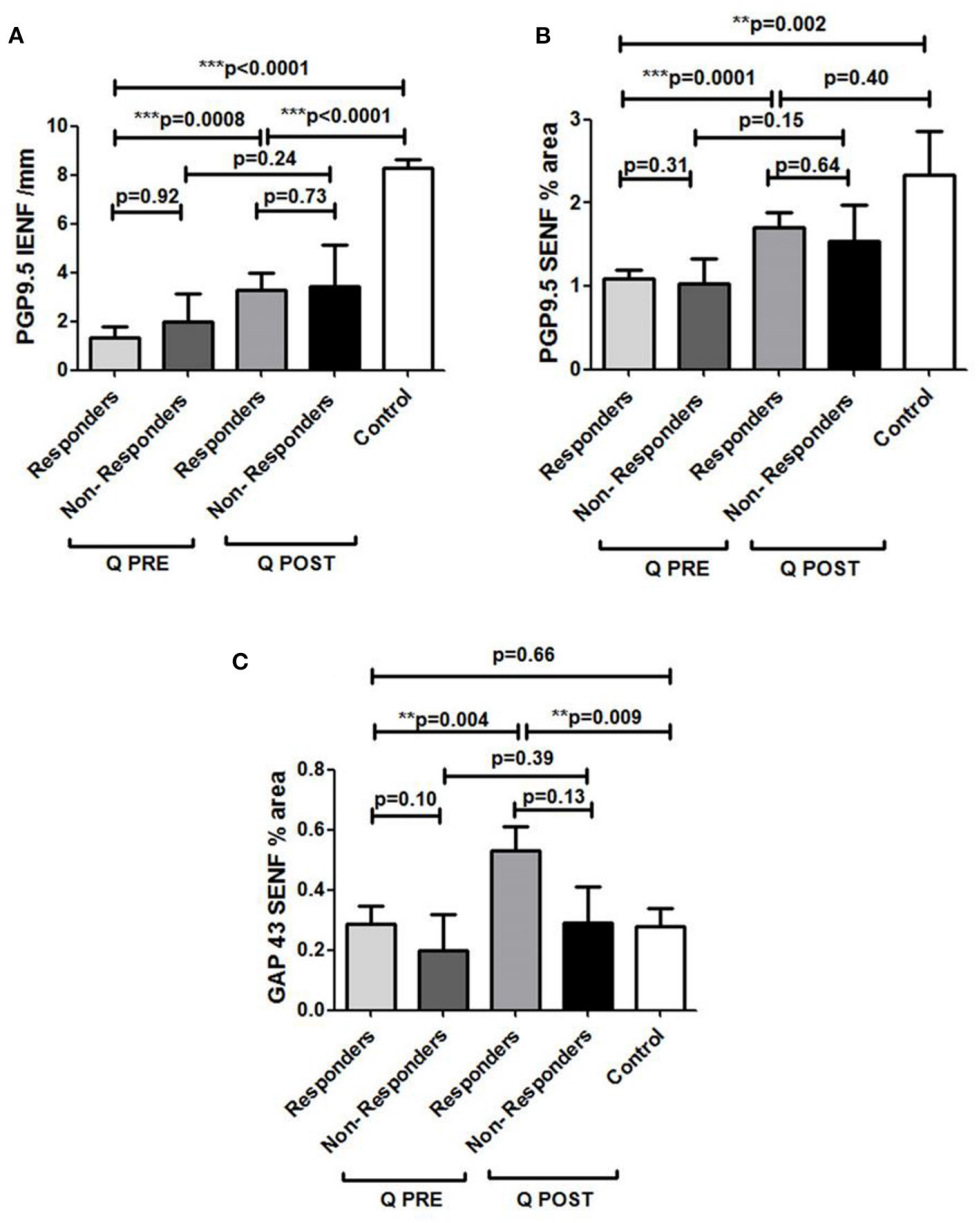
Assessment of skin biopsy in relation to pain relief in Painful Diabetic Peripheral Neuropathy subjects treated with Capsaicin 8% patch. **(A)** Assessment of intra–epidermal PGP9.5 nerve fibers (IENFs/mm; 50 μm sections) in responders (*n* = 18) and non–responders (*n* = 7) at pre–treatment visit (Q PRE) and visit 3 months after treatment (Q POST). Statistically significant increase in PGP9.5 nerve fibers 3 months after capsaicin 8% patch application in responders (***p = 0.0008; paired *t*–test). No statistically significant difference in PGP9.5 nerve fibers 3 months after capsaicin 8% patch application in non–responders' group (*p* = 0.24; paired *t*–test). **(B)** Assessment of sub–epidermal PGP9.5 nerve fibers (SENFs, Area %; 50 μm sections) in responders (*n* = 18) and non–responders (*n* = 7) at pre–treatment visit (Q PRE) and visit 3 months after treatment (Q POST). Statistically significant increase in PGP9.5 nerve fibers 3 months after capsaicin 8% patch application in responders (***p = 0.0001; paired *t*–test). No statistically significant difference in PGP9.5 nerve fibers 3 months after capsaicin 8% patch application in non–responders group (*p* = 0.15; paired *t*–test). **(C)** Assessment of sub–epidermal GAP 43 nerve fibers (SENFs/mm) in responders (*n* = 18) and non–responders (*n* = 7) at pre–treatment visit (Q PRE) and visit 3 months after treatment (Q POST). Statistically significant increase in GAP 43 nerve fibers 3 months after capsaicin 8% patch application in responders (**p = 0.004; paired *t*–test). No statistically significant difference in GAP 43 nerve fibers in non–responder group (*p* = 0.39; paired *t*–test).

PDPN Q + SOC subjects with pain reduction at 3 months had a statistically significant increase in PGP9.5 IENF (^***^p = 0.0008), PGP9.5 SENF (^***^p = 0.0001) in 50 μm thick sections, and GAP43 (^*^p = 0.004), whereas those who reported no pain reduction did not show any increase of PGP9.5 IENF (*p* = 0.24), PGP9.5 SENF (*p* = 0.15) or GAP43 (*p* = 0.39) (Figure 12).

PDPN + Q group showed a positive correlation between the increase of GAP43 regenerating nerve fibers after treatment and the GAP43 nerve fiber density at baseline i.e., before treatment (^**^p = 0.009). There was no significant change in the PDPN + SOC alone group (*p* = 0.81).

Axon-reflex vasodilatation was assessed before and after capsaicin 8% patch application in seven NPDPN + Q group subjects, who showed a positive correlation between the increase of axon-reflex vasodilatation and the increase of PGP9.5 IENF (*p* = 0.03).

## Discussion

The initial aim of this study was to assess the mechanisms of pain relief by the capsaicin 8% patch (Qutenza) in patients with Painful Diabetic Peripheral Neuropathy (PDPN). We aimed to relate any skin biopsy and sensory test changes to the reduction of pain. In view of our initial findings, we then also assessed the efficacy of capsaicin 8% patch (Qutenza) application in improving nerve regeneration in patients with Non-Painful Diabetic Neuropathy (NPDPN).

Reduction of spontaneous (on-going) pain in the PDPN Q+SOC group was significant, and showed a similar time-course, effect-size and responder rates to previous studies (14, 44). As expected, pain relief was significant from week 3 onwards after capsaicin 8% patch (Qutenza) treatment, with some return toward baseline at 3 months. Some patients showed pain relief persisting for 12 months after a single 30-min capsaicin 8% patch application (as reported for pain relief in around 1/3 subjects, persisting at 3 months post-treatment, by Martini et al. (14), though they did not assess skin biopsies). One study patient (see Figures 7, 9) illustrates the potential of capsaicin 8% patch (Qutenza) for both pain relief with disease modification after a single 30-min capsaicin 8% patch application i.e., regeneration and restoration of cutaneous nerve fibers in skin biopsies, collected 3-monthly from treated region over 9 months, alongside significant pain relief.

This increase of nerve fibers was also shown overall in the PDPN Q+SOC group and NPDPN Q+SOC groups at 3 months after capsaicin 8% patch (Qutenza) treatment, for both intra-epidermal (IENFs) and sub-epidermal nerve fibers (SENFs) with the pan-neuronal marker PGP9.5. Sub-epidermal nerve fibers (SENF) assessed with GAP43, a selective marker of regenerating nerve fibers, were also significantly higher 3 months after treatment, and indeed higher than controls. In contrast, these were decreased in the PDPN SOC alone group after 3 months, in comparison with their baseline biopsies. Thermal thresholds for warm perception in the PDPN Q + SOC group were significantly improved 3 months after treatment (*p* = 0.02), and were positively correlated with the improvement in the overall pain score for the Short-Form McGill Pain Questionnaire (*p* = 0.04). Axon-reflex vasodilatation in skin was significantly increased between baseline and 3-month follow-up visit in a subset of subjects tested in the NPDPN Q+SOC group (*p* = 0.006); there was a positive correlation between the increase of axon-reflex vasodilatation and the increase of PGP9.5 IENF (*p* = 0.03).

We are not aware of such an effect on nerve fiber regeneration and density, sensory thresholds, or axon-reflex vasodilatation with any pain or neuropathy treatment reported in PDPN / NPDPN. The patients randomized to PDPN SOC alone in this study showed no significant changes in pain scores, skin biopsies or sensory perception thresholds at the 3-month follow-up visit, other than a decrease of regenerating nerve fibers with GAP43. We have previously reported progressive loss of nerve fibers in skin biopsies collected from patients who had longstanding diabetes, and reported DPN symptoms for a period of 4 years; they had assessments on two visits, with a 6-month interval (24).

The application of high dose capsaicin leads to “defunctionalisation,” a term which encompasses a number of sequential effects, that include capsaicin receptor TRPV1 desensitization, temporary loss of membrane potential, inability to transport neurotrophic factors leading to altered phenotype, and reversible retraction of epidermal and dermal nerve fiber terminals (2). The early stages are akin to deep pruning of nociceptors, which triggers nerve regeneration, and which in turn may be healthier if the milieu is conducive; the latter may explain the prolonged analgesic effect in some patients. The time-course of nerve regeneration following high dose topical capsaicin in human volunteers has been reported by us previously (17), using a range of IENF and SENF nerve markers, including PGP9.5, GAP 43 and TRPV1 (the heat and capsaicin receptor). Regenerating nerve fibers (marked with GAP 43) were prominent in the dermis from week 3 onwards, importantly when significant pain relief is observed following capsaicin 8% patch application in patients, following initial cutaneous terminal degeneration, whereas PGP9.5- and TRPV1-positive IENF were restored later, over months. This is also relevant to the increase of axon-reflex vasodilatation observed, mediated *via* axonal branches in the dermis. Another study in human volunteer thigh skin showed marked loss then incomplete return of IENF density with PGP9.5 at 12 weeks after capsaicin 8% patch application; the IENF density was nearly normal at 24 weeks (18). Experimental studies with repeated low-concentration topical capsaicin applications have investigated intra-epidermal PGP9.5 nerve fiber density (following initial loss) in patients with diabetes, and reported the degree and rate of re-innervation seen in diabetic patients was lower when compared to healthy volunteers (19). Re-innervation in DM subjects was reduced compared to controls, and even further in subjects with DPN. Another study showed a slower reinnervation rate in type 2 DM compared to type 1 DM (20). The depth and degree of “defunctionalization” of cutaneous nerve fibers, and trigger for regeneration, proposed as the mechanism of action for capsaicin 8% patch (Qutenza) (2), may have been different in these studies compared to capsaicin 8% patch (Qutenza), and they did not assess sub-epidermal or regenerating nerve fibers specifically e.g., with GAP43. Notably, we used the high-dose capsaicin 8% (179 mg) patch, applied to the feet (affected/symptomatic region) for 30 min, in diabetic painful and non-painful neuropathy. To our knowledge, no study has been conducted in DPN with skin biopsies taken before and after the treatment to symptomatic feet with the capsaicin 8% (179 mg) patch, or any other topical high-dose capsaicin formulation.

The only similar PDPN studies (19, 20) used a capsaicin dose, formulation, methodology and site of application which was different from our study. They used low concentration topical capsaicin (1.8 g of 0.1% capsaicin), applied for 2 consecutive 24 h periods to the distal thigh, whereas we used capsaicin 8% (179 mg) patch applied to the clinically-affected feet and distal calf for 30 min. The pharmacodynamic effects are therefore likely to be different, based on the proposed mechanisms of action (2), and as discussed below. The mean density of intra-epidermal nerve fibers was higher 3 months after treatment than at baseline in our study, unlike the others, perhaps because of the magnitude of nerve regeneration in some “responders,” which may have resulted from the concentration of capsaicin and dose delivered more swiftly by the capsaicin 8% patch. However, like the other studies, the mean IENF density with PGP9.5 at 3 months post-treatment in our study was still well below the mean value in control subjects (42% of controls for PDPN, and 37% for NPDPN).

Nerve regeneration in patients with diabetes may thus depend on the capsaicin total dose per area, its concentration, delivery time, and skin depth penetration, given our hypothesis that capsaicin induces nerve regeneration *via* a reversible chemical axotomy of cutaneous nerve terminals. In accord, the nerve regeneration we observed in subjects was related to baseline levels of GAP43, suggesting a relationship, or even an endogenous priming of these nerve fibers for the additive regenerative effect of capsaicin. A “priming” effect of on-going regeneration (following degeneration) in affected DPN skin may explain why at 3 months post-treatment the average density is higher than at baseline. It may also explain why in healthy volunteer (normal) thigh skin, the relative lack of this on-going regenerative process pre-treatment may lead to incomplete though overall more robust recovery toward normal values 3 months later (80% of baseline) (18). Further studies are clearly required to explore these issues, including with repeated 2- to 3-monthly applications of Capsaicin 8% patch, which showed progressive pain relief over 12 months in PDPN.

In patients with painful CIPN and NFCI, a 30-min application of capsaicin 8% patch (Qutenza) to the feet and distal calf led to a significant reduction of pain scores, along with a significant increase of intra-epidermal and sub-epidermal nerve fibers. This was accompanied, as studied in CIPN, by an improvement in a range of other mechanistic biomarkers i.e., epidermal levels of Nerve Growth Factor (NGF), Neurotrophin-3 (NT-3), and Langerhans cells (22). Capsaicin 8% patch (Qutenza) treatment in non-freezing cold injury (NFCI) also led to pain relief and nerve regeneration; pain relief correlated with restoration of nerve fibers (23). However, there are some differences between our previous painful CIPN / NFCI and current DPN studies with capsaicin 8% patch (Qutenza). Our previous CIPN study was open label, and while unlikely, as the patients had chronic painful CIPN, spontaneous improvements can occur. The patients were in cancer remission and were not receiving chemotherapy for an average of 2 years when they were treated with capsaicin 8% patch (Qutenza) i.e., they could have improved as part of their natural history. Similarly, the NFCI patients had not been exposed to cold recently. This DPN study is a randomized clinical trial, and the patients all continued to have diabetes with no significant change in its control over the duration of the study pre- and post-capsaicin 8% patch treatment i.e., the cause of the DPN persisted. Hence, we have shown here that capsaicin 8% patch (Qutenza) appears to reverse the natural decline of nerve fiber density (24), and can improve warm sensory perception and axon-reflex vasodilatation, in DPN. We hypothesize that in this study the milieu for the induced regeneration and maturation of nerve fibers was currently more favorable than when the patients first developed DPN, or over the years since. During the 3 months after our capsaicin 8% patch treatment, this milieu, and the propensity of small sensory nerve fibers to regenerate and mature after the trigger of nerve terminal axotomy by capsaicin 8% patch (Qutenza), may have led to disease modification. Repeated 2 to 3-monthly applications of capsaicin 8% patch (Qutenza) may progressively enhance this regenerative process toward normalization; these applications may be required particularly for functional improvements in DPN, and to maintain beneficial effects.

In support of the proposed disease modification mechanism of action: (1) thermal thresholds for warm perception in the PDPN Q + SOC group were significantly improved after 3 months and were positively correlated with the improvement in the overall pain score; (2) PDPN Q + SOC subjects with pain reduction at 3 months had a statistically significant increase in PGP9.5 IENF, SENF and GAP43, whereas those who reported no pain reduction did not; (3) PDPN Q+SOC group showed a positive correlation between the increase of GAP43 regenerating nerve fibers after treatment and the GAP43 nerve fiber density at baseline i.e., propensity for nerve regeneration; (4) NPDPN Q+SOC subjects showed a positive correlation between the increase of axon-reflex vasodilatation and the increase of PGP9.5 IENF; and (5) there was no significant improvement in the PDPN + SOC alone group; on the contrary, there was a decrease of regenerating nerve fibers marked by GAP43.

In future clinical trials, the systematic study of factors that may contribute to or prevent the reported effects of the Capsaicin 8% patch on nerve regeneration would be useful, including concomitant drug regimens. This would require stratification of sufficient numbers of participants e.g., by age, duration and severity of diabetes/neuropathy, concomitant treatments, etc. We have reported here that the density of regenerating nerve fibers at baseline, marked by GAP43, were related to increased nerve regeneration following Capsaicin 8% patch treatment; this marker may thus identify “responders” for nerve regeneration. Other non-invasive markers to identify “responders” would be particularly useful. Future clinical trials should also include systematic studies of the cellular and molecular constituents of the milieu for nerve regeneration, and effect of treatments that that may synergize with Capsaicin 8% patch to enhance nerve regeneration e.g., neurotrophic factors.

Regeneration of sensory nerve fibers and improvement in function in patients with DPN is important clinically, as dysfunction or loss of small sensory nerve fibers including nociceptors are associated with loss of protective sensation, axon-reflex vasodilatation, and trophic changes. These are the major factors for the development and non-healing of foot ulcers, leading to amputations (28, 29). In turn, these complications are associated with a higher risk of mortality (31). Volmer-Thole et al. (32) reported that among all patients with DM the lifetime risk for developing diabetic foot ulceration is 25%, of which the majority will need amputation within four years of initial diagnosis. According to their epidemiological data, solely neuropathy is accountable for about 50% of the cases of diabetic foot syndrome, peripheral arterial occlusive disease on its own is accountable for just 15% of the cases, whereas in 35% a combination of both neuropathy and angiopathy play a role.

As in this study, we have previously reported and discussed an increase of sub-epidermal blood vessels with the same marker vWF in a different cohort of patients with PDPN (45), considered to be secondary to hypoxia and ischemia, also reported in many other tissues from patients with DM (i.e., microangiopathy). In this study, blood vessel density was unchanged after capsaicin 8% patch (Qutenza) treatment, in accord with evidence that it primarily targets nerve fibers. The ratio of nerve fibers to their target organ (including blood vessels and epidermal cells) may play a role in neuropathic pain including PDPN, as we have proposed (2), which the capsaicin 8% patch (Qutenza) may ameliorate.

Diabetic foot syndrome is a major global burden to patients, their careers, and financial resources; better clinical tools and trials will help to improve current treatments outcomes (45, 46). One study (47) estimated that the diabetic foot care cost was between 0.8% to 0.9% of the National Health Service budget for England, attributed mainly to foot ulceration—this was reported to be more than the combined cost of breast, prostate and lung cancers.

## Conclusions

The findings of this study provide an exciting new prospect for the treatment of both painful and non-painful diabetic peripheral neuropathy. It has implications for the prevention of foot ulcers and amputations in patients with DPN, which are an important and increasing complication globally. Loss of sensory nerve fibers or their protective function are the major contributors to the development, recurrence, or non-healing of diabetic foot ulcers, leading to amputations. The capsaicin 8% patch (Qutenza) may provide preventative treatment for the diabetic foot syndrome.

## Data availability statement

The original contributions presented in the study are included in the article/supplementary files, further inquiries can be directed to the corresponding author/s.

## Ethics statement

The studies involving human participants were reviewed and approved by East of England—Cambridgeshire and Hertfordshire Research Ethics Committee. The patients/participants provided their written informed consent to participate in this study.

## Author contributions

PA conceived the study. RP and HF helped with clinical assessments. VM performed the nerve conduction studies. PD analyzed the skin biopsies. VB organized patient referrals and coordination of assessments. All authors contributed to drafting the manuscript and approved the final manuscript.

## Funding

The authors thank Diabetes UK for funding this study. The authors are grateful to Grünenthal GmbH, Germany, who provided supplementary funding for Capsaicin 8% patch Qutenza studies.

## Conflict of interest

This study received supplementary funding from Grünenthal GmbH. The funder had the following involvement with the study: interpretation of data. PA has received symposia speaker fees and advisory board honoraria from Grunenthal, but no personal remuneration for conducting this study.

The remaining authors declare that the research was conducted in the absence of any commercial or financial relationships that could be construed as a potential conflict of interest.

## Publisher's note

All claims expressed in this article are solely those of the authors and do not necessarily represent those of their affiliated organizations, or those of the publisher, the editors and the reviewers. Any product that may be evaluated in this article, or claim that may be made by its manufacturer, is not guaranteed or endorsed by the publisher.
